# A bibliometric and knowledge-map analysis of the glymphatic system from 2012 to 2022

**DOI:** 10.3389/fnmol.2023.1148179

**Published:** 2023-08-28

**Authors:** Changkai Hou, Wen Ren, Bangyue Wang, Xi’an Fu, Quanlei Liu, Jian Li, Hao Zhang, Weihan Wang, Xinyu Yang, Penghu Wei, Guoguang Zhao

**Affiliations:** ^1^Department of Neurosurgery, Xuanwu Hospital of Capital Medical University, Beijing, China; ^2^Department of Radiology, The Affiliated Suzhou Hospital of Nanjing Medical University, Suzhou, Jiangsu, China; ^3^Department of Neurosurgery, Tianjin Medical University General Hospital, Tianjin, China; ^4^Department of Neurosurgery, The Affiliated Suzhou Hospital of Nanjing Medical University, Suzhou, Jiangsu, China; ^5^Department of Neurosurgery, Nanjing University Medical School Affiliated Nanjing Drum Tower Hospital, Nanjing, China; ^6^Department of Neurosurgery, Beijing Friendship Hospital, Capital Medical University, Beijing, China

**Keywords:** glymphatic system, CiteSpace, VOSviewer, bibliometrics, visualization, WoSCC

## Abstract

**Objective:**

To explore the development context, research hotspots and frontiers in the glymphatic system (GS) field from 2012 to 2022 by bibliometric analysis.

**Methods:**

The Web of Science Core Collection (WoSCC) database was searched for articles published between 2012 and 2022. Microsoft Excel was used to manage the data. VOSviewer, CiteSpace, GraphPad Prism, the Web of Science, and an online analysis platform for bibliometrics (http://bibliometric.com/) were used to analyze the countries, institutions, journals, and collaboration networks among authors and the types of articles, developmental directions, references, and top keywords of published articles.

**Results:**

A total of 412 articles were retrieved, including 39 countries/regions, 223 research institutes and 171 academic journals. The subject classifications related to the GS were Neuroscience, Clinical Neuroscience and Radiology/Nuclear Medicine/Medical Imaging. The United States has maintained its dominant and most influential position in GS research. Among research institutions and journals, the Univ Rochester and Journal of Cerebral Blood Flow and Metabolism had the highest number of academic articles, respectively. Nedergaard M had the most published article, and Iliff JJ had the most co-citations. The top two keywords with the highest frequency were “glymphatic system” and “cerebrospinal fluid.”

**Conclusion:**

This research provides valuable information for the study of the GS. The bibliometric analysis of this area will encourage potential collaborations among researchers, defining its frontiers and directions for development.

## Introduction

As an important research discovery in the field of neuroscience, the glymphatic system (GS) has received extensive attention in recent years (Iliff et al., 2012; Rasmussen et al., 2018; Mestre et al., 2020; Nedergaard and Goldman, 2020). The initial literature indicated that the GS participated in the pathophysiological changes of many nervous system diseases, such as neurodegenerative diseases, by regulating the transport of interstitial metabolic wastes (Iliff et al., 2012; Harrison et al., 2020; Reeves et al., 2020). As far as we know, the function of the GS is related to age, the circadian rhythm, exercise, the heart rhythm, and the presence of degenerative disease, stroke, trauma or other conditions, which suggests that the GS has great research potential in the field of neuroscience (Goulay et al., 2017; He et al., 2017; Wang et al., 2017; Hablitz et al., 2020; Hou et al., 2022a,b).

Since the concept of the GS was proposed in 2012, an increasing number of relevant studies have been published in the past decade. However, to date, no research has summarized and analyzed the literature in this field using bibliometric methods. For the first time, this study used the bibliometric method to review and visually analyze the original works on the GS published in the past decade and predicted future research trends. We expect that the findings of this study can provide an overall understanding for researchers interested in the GS and provide new clues for subsequent research.

## Materials and methods

### Data collection

**Figure 1:**
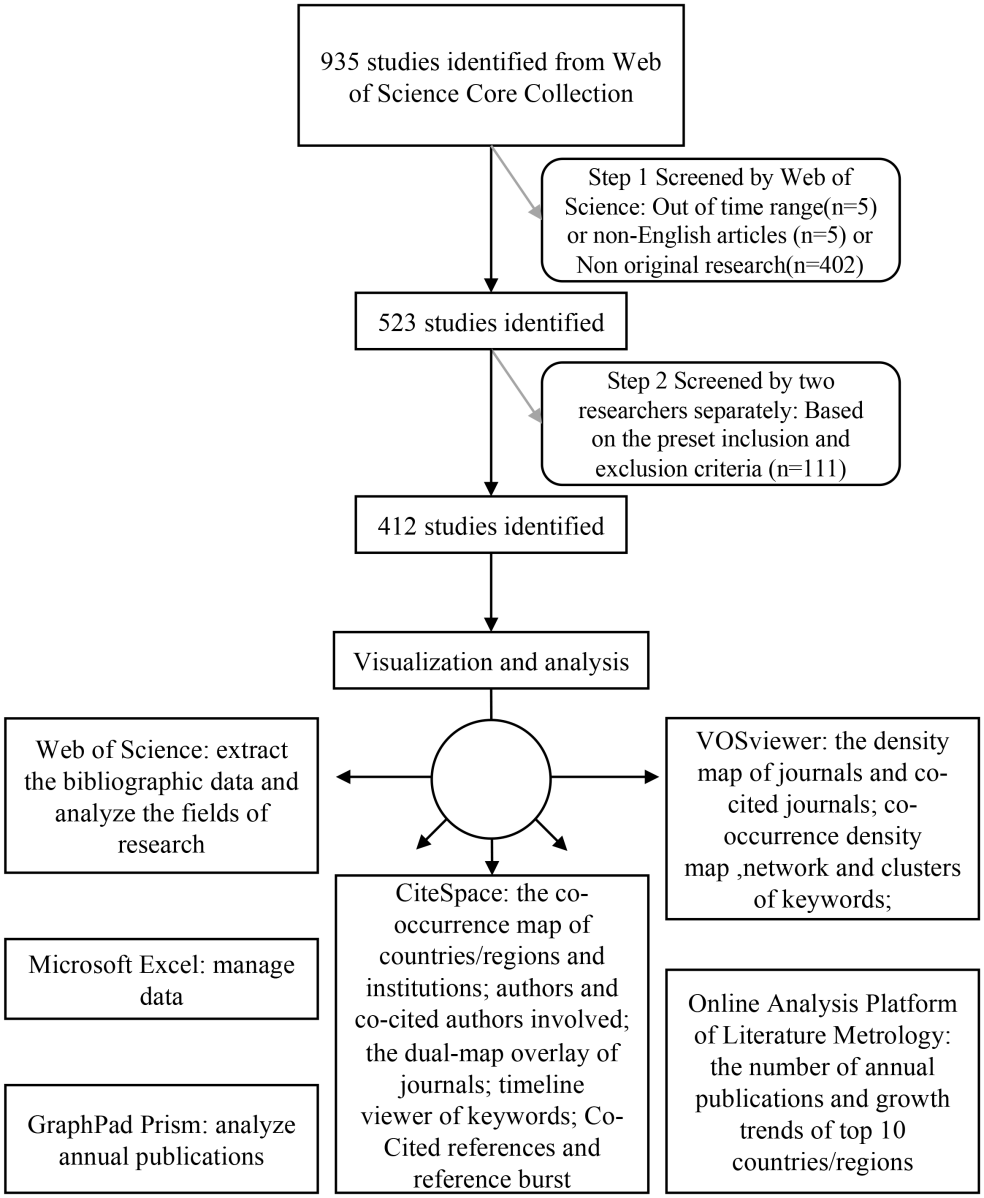
Study flow diagram.

Journal publications have strong timeliness and can accurately reflect the progression of and changes in a research topic. In this study, the scientific literature database Web of Science Core Collection (WoSCC) was used to search relevant literature, as it is among the largest and most comprehensive electronic scientific literature databases worldwide. A flow chart of research inclusion is shown in Figure 1. The retrieval strategy used in this study was set to TS = (“glymphatic system” OR “glymphatic pathway” OR “Glymphatic Pathways” OR “Pathway, Glymphatic” OR “Glymphatic Clearance System” OR “glymphatic OR “glial lymphatic” OR “Brain Perivascular Spaces” OR “Brain Perivascular Space” OR “Perivascular Space, Brain” OR “paravascular pathway”). The retrieval time range was from 2012 to 24 September 2022. The language was limited to English. A total of 925 articles were initially obtained. After the article type was confined to articles, the number of documents was reduced to 523. The articles were further screened according to the following criteria.

### Inclusion and exclusion criteria

The following inclusion criteria were used to review and screen the preliminarily identified publications after the initial search: (1) the publication language was limited to “English”; (2) the publication type was restricted to “article”; (3) the publication was collected from the WoSCC Citation Index Expanded (SCI-E) database; and (4) the time span was from 2012 to 2022; (5) for the research content of the selected version, the research index should include the GS or the space around blood vessels. To avoid the deviation caused by daily database updating, all article retrieval and data collection steps were completed on the same day. According to the inclusion and exclusion criteria, 412 articles were finally included in our study.

### Data analysis

All data included by the defined criteria were collected from the WoSCC database. The downloaded files were imported into Microsoft Excel to manage the data. The analysis of annual publications was assessed by GraphPad Prism. The Web of Science was used to analyze the field of research. The online analysis platform for bibliometrics^1^ provided the bibliometric analysis of the number of publications by country in different years and cooperation between countries. VOSviewer was used to draw the density map of journals and cocited journals and to analyze keyword co-occurrence and network clustering to obtain the density map and network map. CiteSpace was used to analyze and visualize the co-occurrence of authors, cocited authors, institutions and countries/regions, the double graph superposition of published journals and the timeline graph of keywords. In addition, CiteSpace analyzed and counted cocited literature and references.

## Results

### Annual publication growth trend

**Figure 2:**
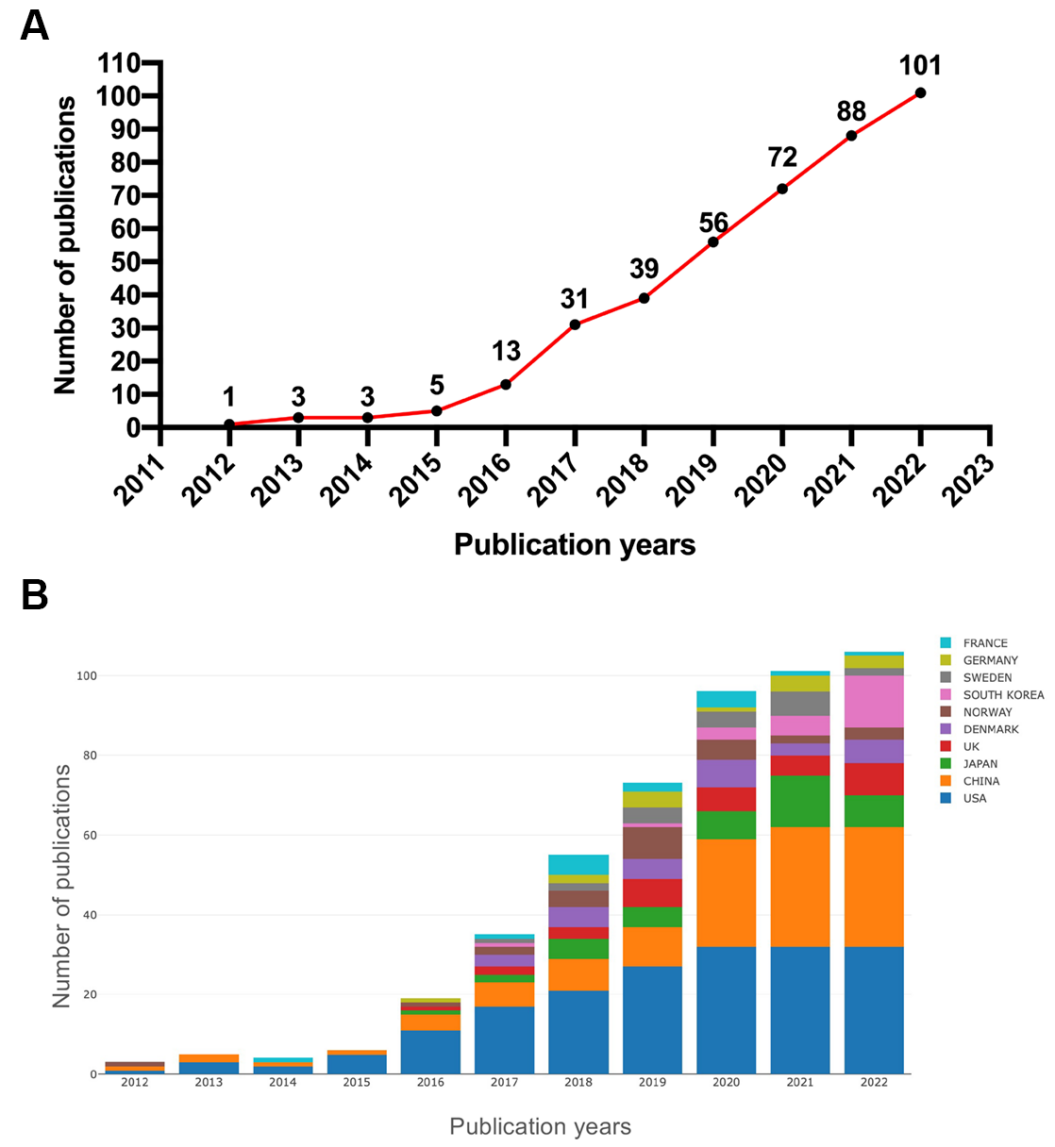
**(A)** Annual publication trends in the glymphatic system from 2012 to 2022; **(B)** overview of annual publication changes in the top 10 countries in the GS field from 2012 to 2022.

To obtain the development trend of relevant research in the GS field, the 412 articles finally included were stratified by publication year. The original research in this field has increased year by year since the concept of the GS was proposed in 2012 (Figure 2). During the time period from 2012 to 2015, although the output of publications was small, these literatures play an important role in the concept initiation of GS, which laid the research foundation for the GS and was of great significance. Strikingly, the number of relevant papers grew rapidly from 2015 to 2021. At the time of this study, by September 24th, 101 related papers were published in 2022, which exceeds the total from 2021. This indicates that the research on GS has gradually attracted the interest of scholars after a certain period of accumulation. Collectively, the research on the GS is still in its infancy, and the number of studies is gradually showing a surge. It is foreseeable that more original research will be published in the coming years. Although the current research results have confirmed the scientific value and development prospects of GS, these results are insufficient to support the mature application of this field from theory to clinical practice, and more original research is urgently needed.

### Research fields

**Figure 3:**
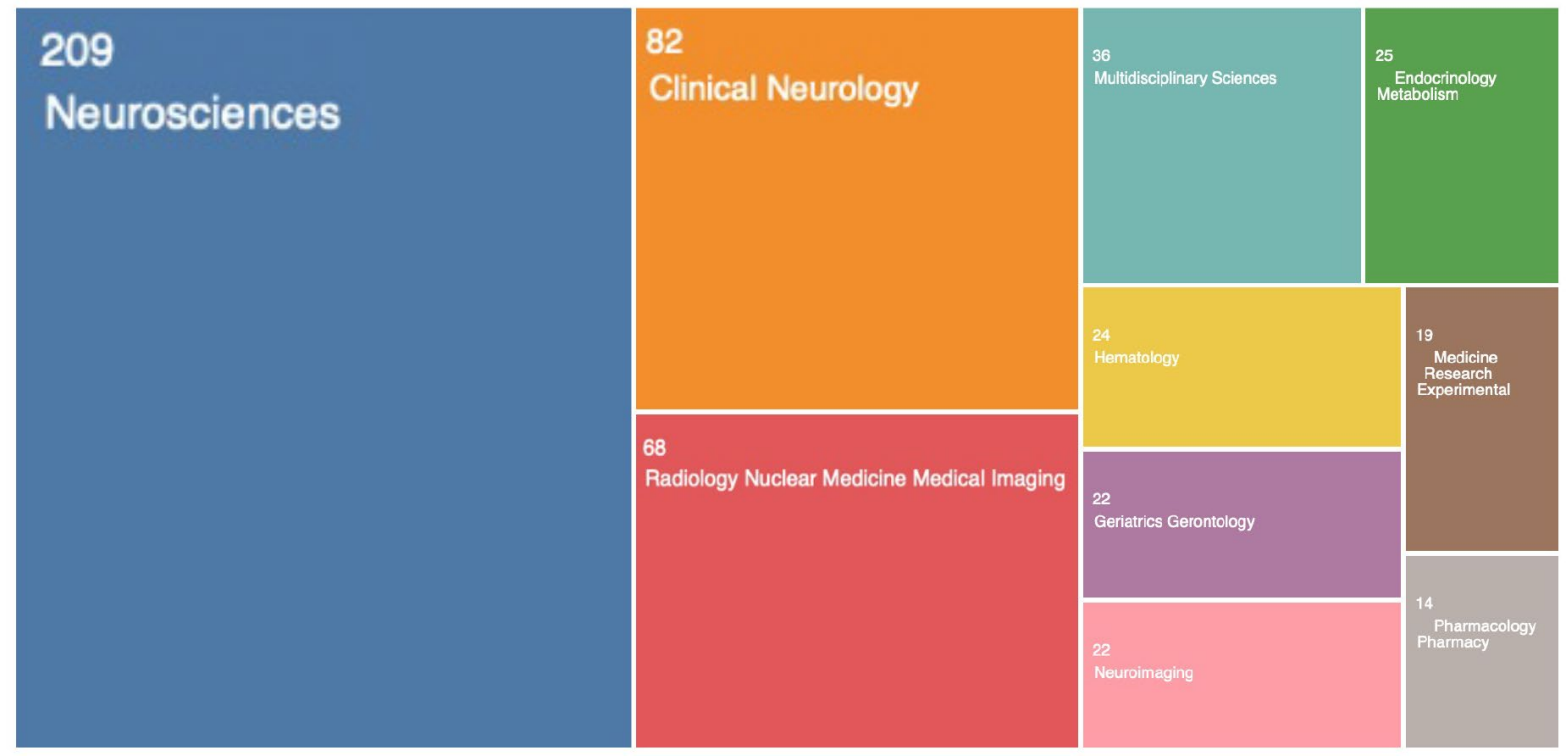
Top 10 glymphatic system research fields from 2012 to 2022.

Web of Science was used to analyze the fields of research. The top 10 research fields were ranked (Figure 3). Notably, Neuroscience, Clinical Neurology, and Radiology/Nuclear Medicine/Medical Imaging were among the top 3 fields in terms of GS research, with Neuroscience (209/50.7%) ranking first, followed by Clinical Neurology (82/19.9%) and Radiology/Nuclear Medicine/Medical Imaging (68/16.5%). Therefore, we can roughly understand that previous studies on the GS focused on basic research, clinical translation and imaging evaluation.

### Countries/regions and institutions

**Figure 4:**
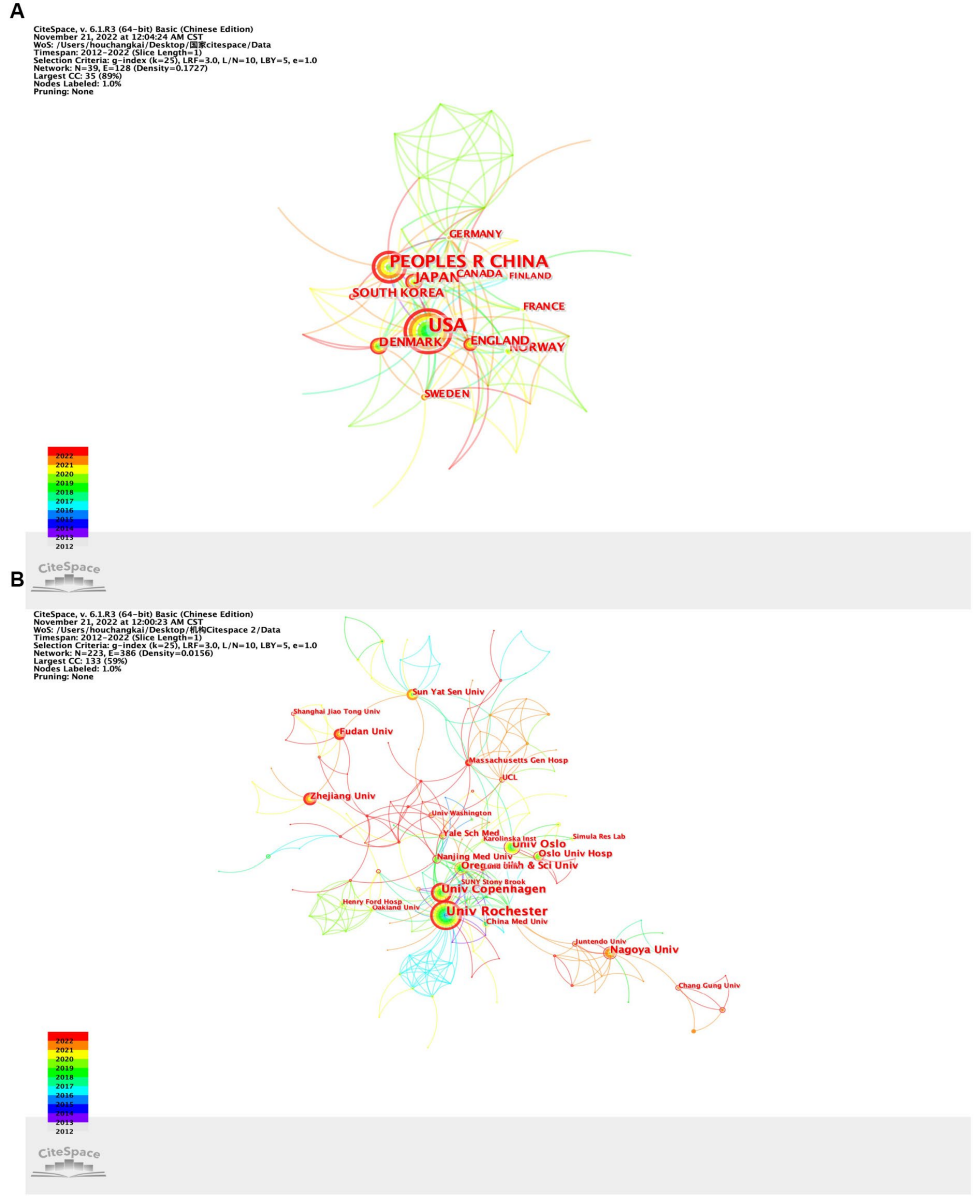
**(A)** Collaboration network of countries/regions; **(B)** collaboration network of institutions.

According to the number of articles published each year, the most active countries/regions and institutions in the GS research field were summarized (Figure 4). Thirty-nine countries/regions and 223 research institutes have contributed research to the GS field. It should be noted that the country with the largest output of publications was the United States (*n* = 187, 45.4%), followed by China (*n* = 123, 29.9%), Japan (*n* = 41, 10.0%), Denmark (*n* = 29, 7.0%) and Norway (*n* = 26, 6.3%; Figure 4A; Table 1). Subsequently, we filtered and visualized those countries, and constructed a collaborative network based on the number and relationship of publications in each country (Figure 4A). Notably, there is a lot of active cooperation between different countries. For example, the United States has active cooperation with England, Denmark, and China, China has close cooperation with the Norway, Finland, the United States and Germany. Significantly, most of the top ten countries are developed countries. China has also provided a large number of research articles with the increase in scientific research investment and attention in recent years. The top three central countries were the United States (0.44), England (0.27) and Germany (0.23) (Supplementary Table S1). By definition, a high centrality indicates that the centrality index is greater than 0.1, indicating that the node plays an important role in the target domain. The above data indicated that these three countries played an important role in international cooperation and exchange.

**Table 1:** Top 10 countries/institutions in terms of publications.

Rank	Count	Country/Region	Centrality	Rank	Count	Institution	Centrality
1	187	United States	0.44	1	44	Univ Rochester (United States)	0.30
2	123	People’s Republic of China	0.21	2	26	Univ Copenhagen (Denmark)	0.07
3	41	Japan	0.03	3	22	Nagoya Univ (Japan)	0.10
4	29	Denmark	0.07	4	22	Univ Oslo (Norway)	0.09
5	26	Norway	0.01	5	16	Oregon Hlth and Sci Univ (United States)	0.04
6	25	England	0.27	6	14	Fudan Univ (China)	0.04
7	24	South Korea	0.00	7	13	Zhejiang Univ (China)	0.04
8	20	Sweden	0.13	8	13	Oslo Univ Hosp (Norway)	0.00
9	16	Germany	0.23	9	12	Nanjing Med Univ (China)	0.07
10	15	France	0.02	10	12	Sun Yat Sen Univ (China)	0.11

In addition, the top 10 institutions that published articles were listed (Table 1). Univ Rochester (*n* = 44, 10.7%) ranked first, followed by Univ Copenhagen (*n* = 26, 6.3%), Nagoya Univ (*n* = 22, 5.3%), Univ Oslo (*n* = 22, 5.3%) and Oregon Health and Sci Univ (*n* = 16, 3.9%; Figure 4B; Table 1). Among them, Univ Rochester (0.30), Massachusetts Gen Hosp (0.18) and Keio Univ (0.14) showed high centrality, which means that these institutions occupy an important position in research in the GS field (Supplementary Table S1). Subsequently, we filtered and visualized those institutions, and constructed a collaborative network based on the number and relationship of publications of each institution (Figure 4). As shown in Figure 4B, the cooperation between Univ Rochester, Oregon Health and Sci Univ and Univ Copenhagen is very close. In addition, we note that Nagoya Univ、Sun Yat Sen Univ、Fudan Univ and Zhejiang Univ, although they publishes lots of papers, have not close cooperation partnership with principal organs of GS.

As shown, there were most cyan and yellow links between countries around 2020 (Figure 4A), which indicated that cooperation among countries was most popular in 2020. The reduction in cooperation in the last 2 years may be related to the reduction in international exchanges caused by the epidemic situation. There were many cyan, red and yellow links among agencies (Figure 4B), which indicated that cooperation among agencies was intensive in recent years.

### Visual analysis of journals and co-cited journals

**Figure 5:**
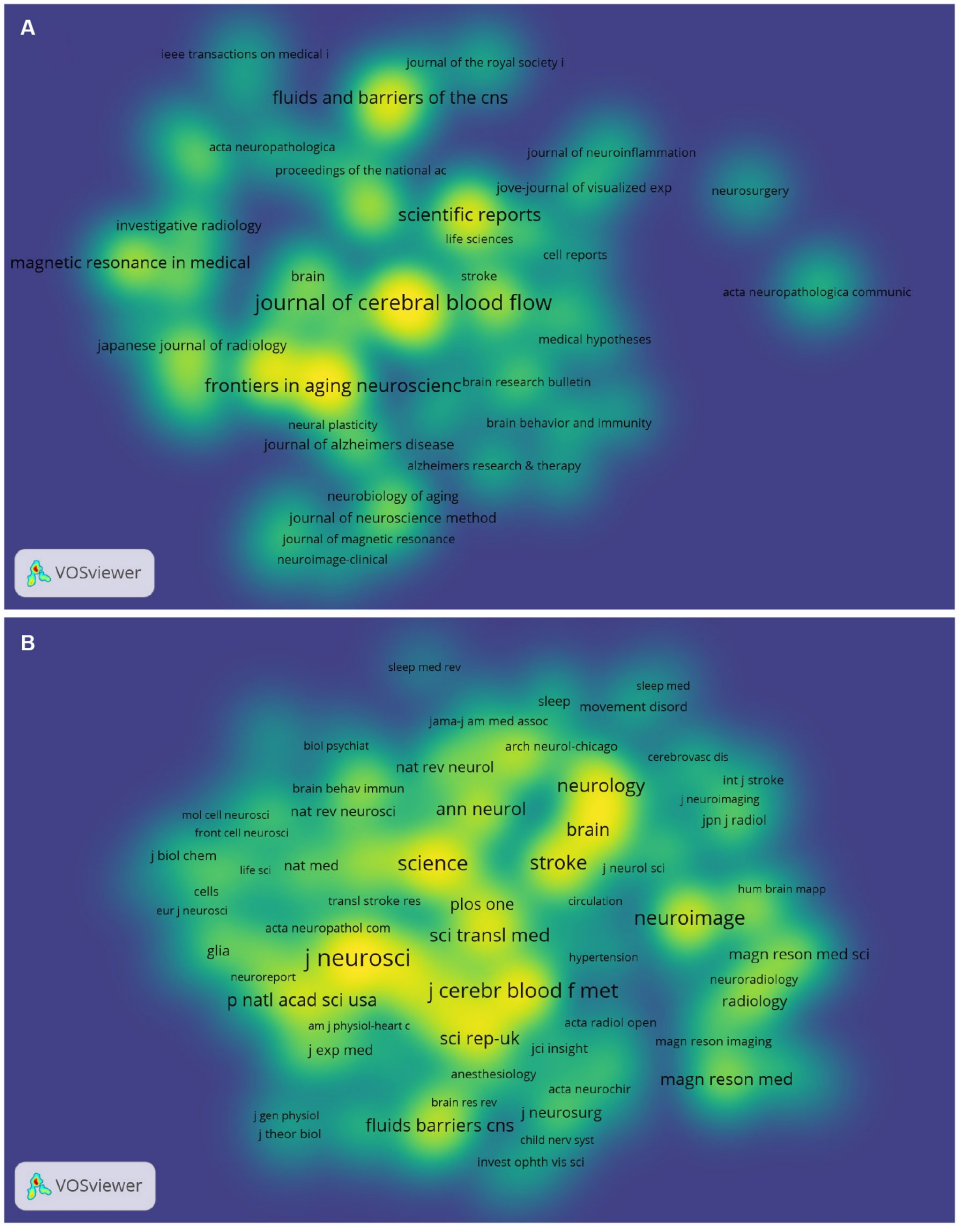
Density map of journals **(A)** and cocited journals **(B)** in GS research. **(A)** shows journals with ≥2 publications (72 journals in total); **(B)** shows journals with ≥20 citations (162 journals in total).

VOSviewer was used to analyze co-cited and cited journals. The results showed that 412 papers were published in 171 academic journals. The journal that published the most papers was the Journal of Cerebral Blood Flow and Metabolism (*n* = 24), followed by Frontiers in Aging Neuroscience (*n* = 16), Scientific Reports (*n* = 16), Fluids and Barriers of the CNS (*n* = 15) and Frontiers in Neuroscience (*n* = 15) (Table 2). Among the top 10 journals, eight published more than 10 papers, and seven were located in the Q1 Journal Citation Report (JCR) division. Neuroimage (IF = 7.4) had the highest impact factor (IF). Journal co-citation analysis revealed the interdependence and cross-relationship between them. The Journal of Neuroscience was cited 645 times, ranking first, followed by the Journal of Cerebral Blood Flow and Metabolism (484 times) and Science (451 times). The top 10 cited journals all belonged to the Q1 JCR division, and the IF of Science was the highest (IF = 63.714). The density map can visually display the published journals and co-cited journals (Figure 5).

**Table 2:** The top 10 journals distributed by publications and citations.

Rank	Journal	Count	IF(2021)	JCR(2021)	Rank	Co-cited journal	Citation	IF(2021)	JCR(2021)
1	Journal of cerebral blood flow and metabolism	24	6.96	Q1	1	Journal of neuroscience	645	6.709	Q1
2	Frontiers in aging neuroscience	16	5.702	Q1	2	Journal of cerebral blood flow and metabolism	484	6.96	Q1
3	Scientific reports	16	4.996	Q1	3	Science	451	63.714	Q1
4	Fluids and barriers of the cns	15	6.961	Q1	4	Stroke	422	10.17	Q1
5	Frontiers in neurology	15	4.086	Q2	5	Neuroimage	409	7.4	Q1
6	Magnetic resonance in medical sciences	11	2.76	Q3	6	Science translational medicine	339	19.319	Q1
7	Neuroimage	11	7.4	Q1	7	Neurology	316	11.8	Q1
8	Journal of neuroscience	10	6.709	Q1	8	Proceedings of the national academy of sciences of the united states of america	298	9.58	Q1
9	Magnetic resonance in medicine	9	3.737	Q1	8	Annals of neurology	281	11.274	Q1
10	American journal of neuroradiology	6	4.966	Q2	10	Scientific reports	277	4.996	Q1

**Figure 6:**
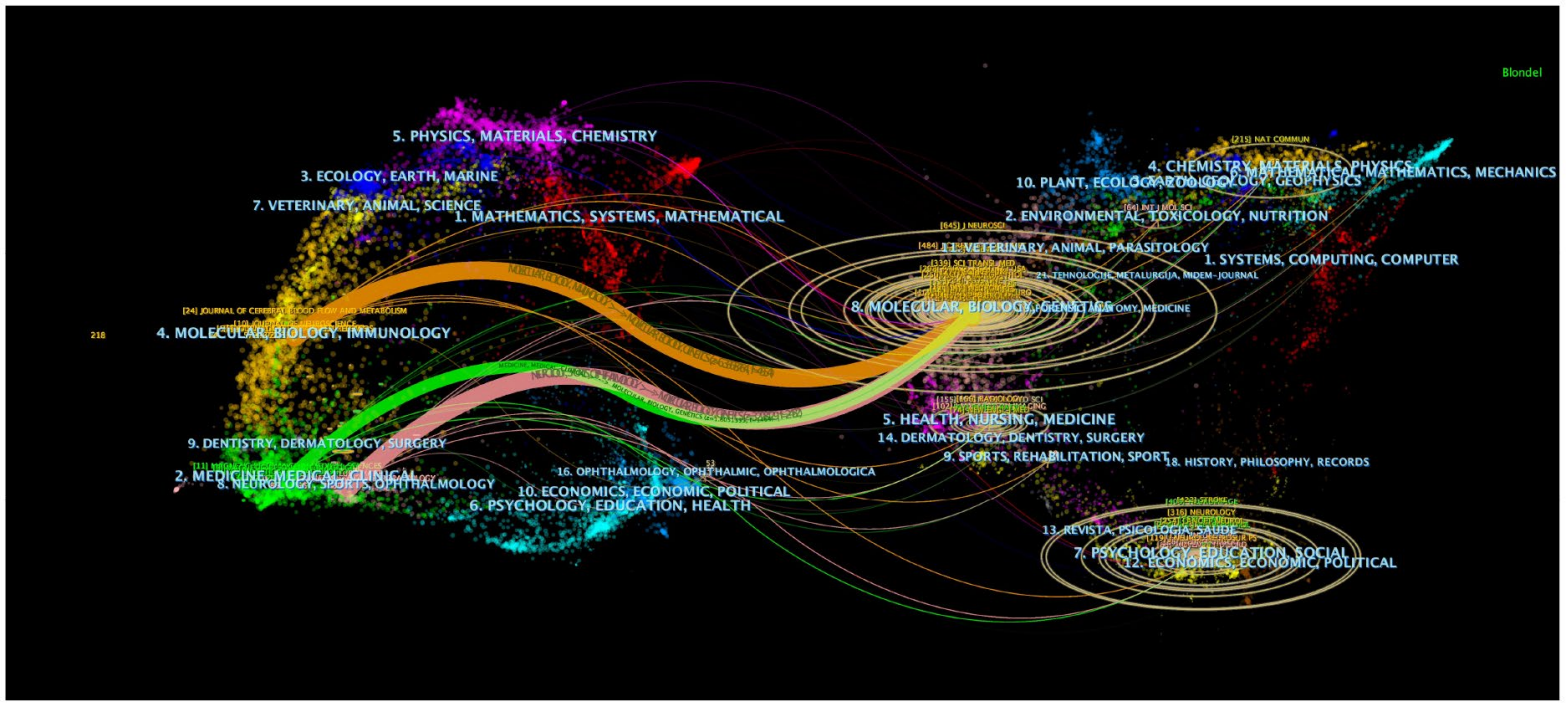
A dual-map overlay of journals related to research on the GS.

The dual-map overlay of journals can intuitively show the distribution of journals in various disciplines and the evolution of citation trajectories. The dual-map overlay of journals in Figure 6 demonstrates the topic distribution of the journals. The color path represents the cited relationship. The left shows the citing journals, and the right shows the cited journals. Three main reference paths are shown (Figure 6). The present research indicated that papers published in the journal “Molecular/Biology/Genetics” were often cited by “Molecular/Biology/Immunology,” “Medical/Medicine/Clinical” and “Neurology/Sports/Ophthalmology.”

### Analysis of authors and co-cited authors

**Figure 7:**
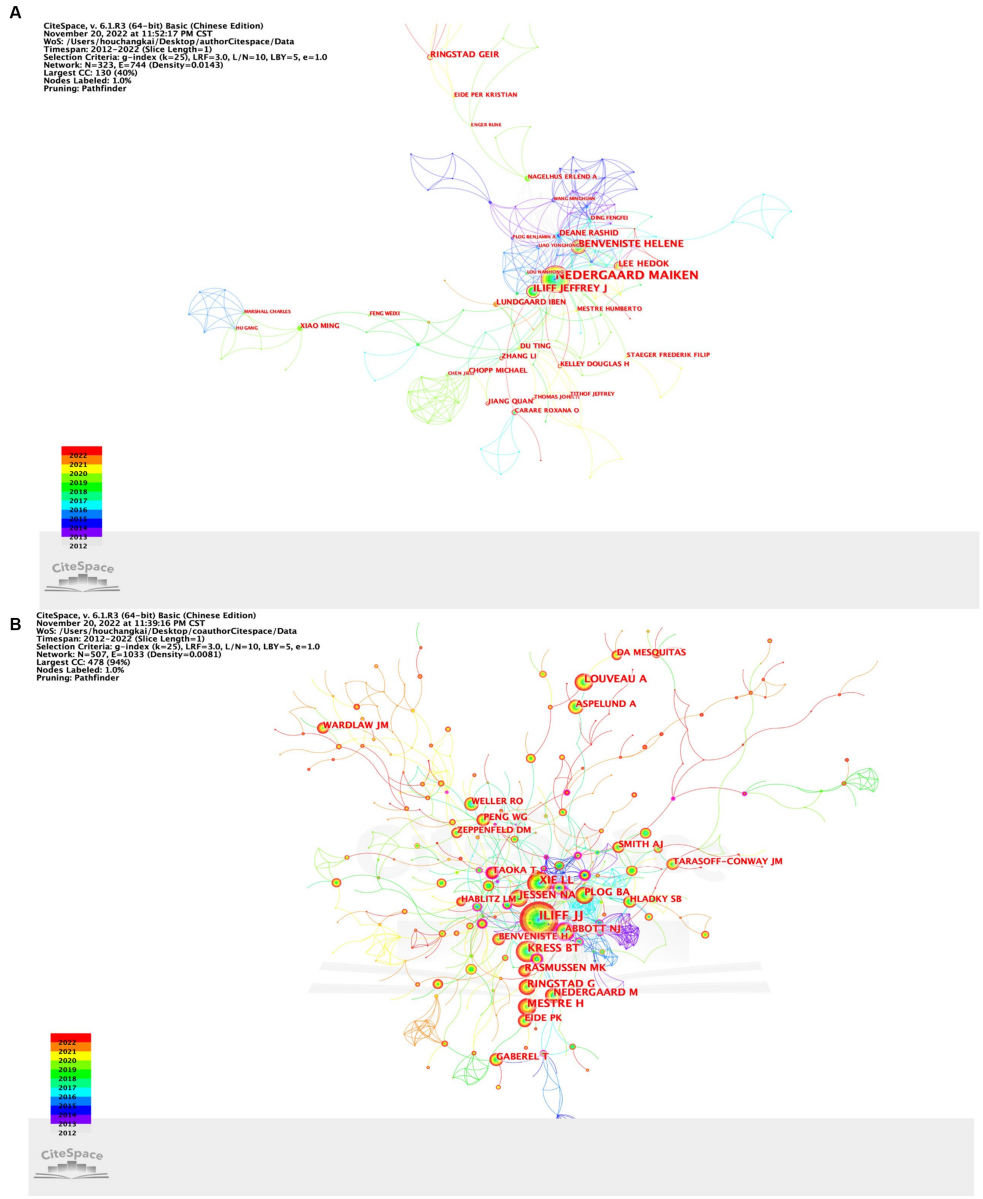
**(A)** CiteSpace visualization map of authors involved in research on the GS; **(B)** CiteSpace visualization map of cocited authors involved in research on the GS.

Our results suggested that the 412 selected publications were produced by 324 authors. As listed in Table 3, Nedergaard M headed 42 documents, followed by Benveniste H with 16 documents, Ilff JJ with 12 documents, Lee H with 11 documents and Park KM with 11 documents. It should also be noted that Nedergaard M showed the highest centrality among the top 10 authors, which was 0.17. The others had lower centrality, which means that these authors have great potential for improving collaboration. Co-cited authors were defined as the literature of two authors being cited in one or more papers by the third author at the same time. The co-cited author analysis could intuitively reveal the research strength and hot topics. The most frequently co-cited author was Ilff JJ (*n* = 339), followed by Xie LL (*n* = 162), Mestre H (*n* = 150), Kress BT (*n* = 145) and Louveau A (*n* = 117). The centrality of the top 10 co-cited authors was not high (Table 3). As shown, there was a certain degree of cooperation between different authors (Figure 7). Each circle represents an author, and the lines between circles represent cooperation between authors. The thicker lines indicate closer cooperation, and different colors represent different years. As shown in authors/co-citation authors network graphs (Figures 7A,B), Nedergaard M, Benventite H and Ilff JJ have the largest nodes because they publish the most related publications. Besides, we observed close collaboration among multiple authors. For example, Ilff JJ, Kress BT, and Xie LL has active cooperation with Nedergaard M, etc.there are also active collaborations among different co-cited authors, such as Ilff JJ and Kress BT, Xie LL, and Plog BA.

**Table 3:** The top 10 authors and co-cited authors involved in research on glymphatic system.

Rank	Author	Count	Centrality	Rank	Co-cited Author	Count	Centrality
1	Nedergaard Maiken	42	0.17	1	Iliff Jeffrey J	339	0.05
2	Benvensite Helene	16	0.05	2	Xie LL	162	0.06
3	Iliff Jeffrey J	12	0.06	3	Mestre H	150	0.02
4	Lee Hedok	11	0.00	4	Kress BT	145	0.04
5	Park Kang Min	11	0.00	5	Louveau A	117	0.03
6	Naganawa Shinji	10	0.00	6	Jessen NA	111	0.00
7	Ringstad Geir	10	0.01	7	Ringstad Geir	110	0.03
8	Taoka Toshiaki	10	0.00	8	Plog BA	106	0.03
9	Deane Rashid	9	0.04	9	Martin Kaag Rasmusssen	91	0.02
10	Lundgaard Iben	8	0.01	10	Nedergaard Maiken	91	0.01

### Co-cited references and reference bursts

CiteSpace was used to search the top 10 most commonly cited references. The top 10 co-cited references with strong bursts related to the GS were recorded (Table 4). These 11 references have all been cited more than 50 times. Of note, 10 of them were derived from the Q1 division. The top 3 references were entitled “The glymphatic pathway in neurological disorders,” “Glymphatic MRI in idiopathic normal pressure hydrocephalus” and “Flow of cerebrospinal fluid is driven by arterial pulsations and is reduced in hypertension “. Compared to new articles, older articles tend to accumulate more citations. In order to evaluate the relevance of the literature in this field more equitably, we analyzed the top 10 most frequently co-cited references (Supplementary Table S2) based on the number of times the literature is cited each year. Most of the 10 articles are cited more than 10 times per year, and 7 overlap with Table 4. The first ranked document is still Martin Kaag Rasmussen et al.’s “The glymphatic pathway in neurological disorders”.

**Table 4:** The top 10 co-cited references related to the glymphatic system field.

Rank	Year	Author	Title	Journal	Citation	Centrality
1	2018	Martin Kaag Rasmussen	The glymphatic pathway in neurological disorders	Lancet Neurology	88	0.01
2	2017	Geir Ringstad	Glymphatic MRI in idiopathic normal pressure hydrocephalus	Brain	73	0.11
3	2018	Humberto Mestre	Flow of cerebrospinal fluid is driven by arterial pulsations and is reduced in hypertension	Nature communication	68	0.02
4	2014	Benjamin T Kress	Impairment of paravascular clearance pathways in the aging brain	Annals Of Neurology	67	0.20
5	2018	Humberto Mestre	Aquaporin-4-dependent glymphatic solute transport in the rodent brain	Elife	67	0.03
6	2015	Nadia Aalling Jessen	The Glymphatic System: A Beginner’s Guide	Neurochemical Research	66	0.01
7	2015	Antoine Louveau	Structural and functional features of central nervous system lymphatic vessels	Nature	64	0.05
8	2018	Benjamin A Plog	The Glymphatic System in Central Nervous System Health and Disease: Past, Present, and Future	Annual Review Of Pathology	57	0.02
9	2018	Geir Ringstad	Brain-wide glymphatic enhancement and clearance in humans assessed with MRI	JCI Insight	56	0.02
10	2017	Toshiaki Taoka	Evaluation of glymphatic system activity with the diffusion MR technique: diffusion tensor image analysis along the perivascular space (DTI-ALPS) in Alzheimer’s disease cases	Japanese Journal of Radiology	54	0.05
10	2014	Jeffrey J Iliff	Impairment of glymphatic pathway function promotes tau pathology after traumatic brain injury	Journal of Neuroscience	54	0.09

**Figure 8:**
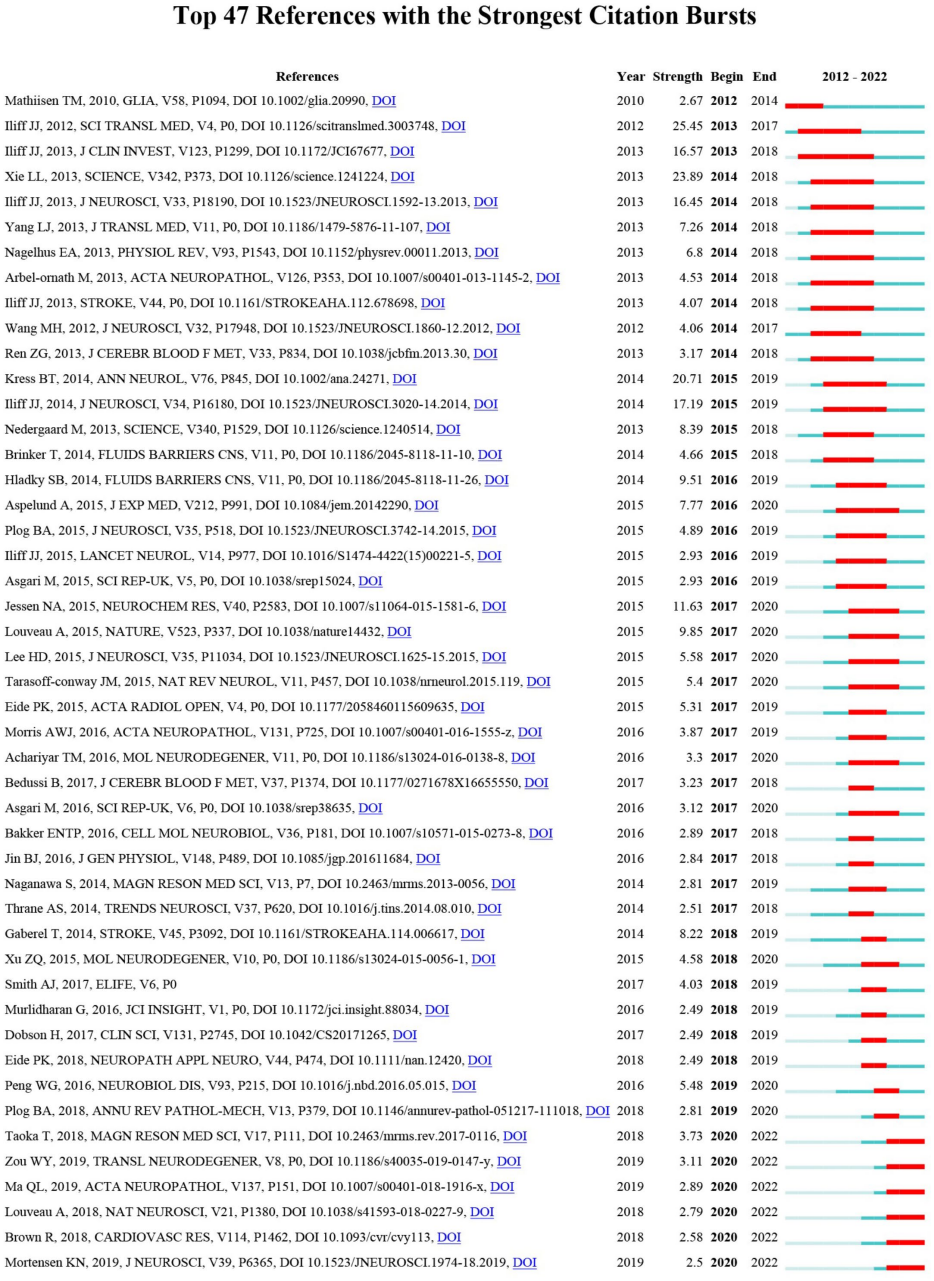
The top 47 references with the strongest citation bursts involved in GS. The blue bars indicate that the reference has been published; the red bars indicate citation burstness.

When the citations of certain documents suddenly increase during a certain period, the document is referred to as a reference with intense citation bursts, which can provide helpful insight for discovering emerging topics and research focus in a certain field. This study obtained 47 references with the most powerful citation bursts. All are shown in Figure 8. The title of the top three most cited references was “A paravascular pathway facilitates CSF flow through the brain parenchyma and the clearance of interstitial solutes, including amyloid β” (Strength: 25.45; year of publication: 2012), “Sleep drives metabolite clearance from the adult brain “(Strength: 23.89; year of publication: 2013) and “Impairment of paravascular clearance pathways in the aging brain “(Strength: 20.71; year of publication: 2014). Figure 8 shows that the citation period of more than 10 articles exceeded 4 years, which indicated that these articles gained more lasting attention. Six articles were still in the period of citation outbreak, which can represent the current and future GS research directions to some extent.

### Keyword co-occurrence, clustering and evolution

Keyword co-occurrence, clustering and evolution can reflect the emerging concepts that increased abruptly over time. Keywords were visualized to further understand the research focus and direction in this field. A total of 1751 keywords were extracted. AQP4, AQP-4, aquaporin-4 and aquaporin 4 were merged because they have the same meaning. The top 20 keywords with the largest number of occurrences are shown in Table 5. Our results suggested that “glymphatic system” and “cerebrospinal fluid” were the most prominent terms, with 177 and 131 co-occurrences, respectively, followed by clearance (*n* = 120), brain (*n* = 113) and magnetic resonance imaging (*n* = 107). These words represented the hotspots in the GS research field. The density map of keywords can intuitively display these high-frequency keywords.

**Table 5:** The top 20 keywords related to glymphatic system.

Rank	Keyword	Count	Rank	Keyword	Count
1	Glymphatic system	177	11	System	63
2	Cerebrospinal-fluid	131	12	Glymphatic	49
3	Clearance	120	13	Perivascular spaces	47
4	Brain	113	14	Central-nervous-system	40
5	Magnetic resonance imaging	107	15	Amyloid beta	35
6	Alzheimer’s disease	98	16	Mouse model	35
7	Pathway	91	17	Virchow-robin spaces	35
8	Aquaporin 4	75	18	Dementia	34
9	Interstitial fluid	69	19	Glymphatic pathway	34
10	Impairment	65	20	Small vessel disease	32

**Figure 9:**
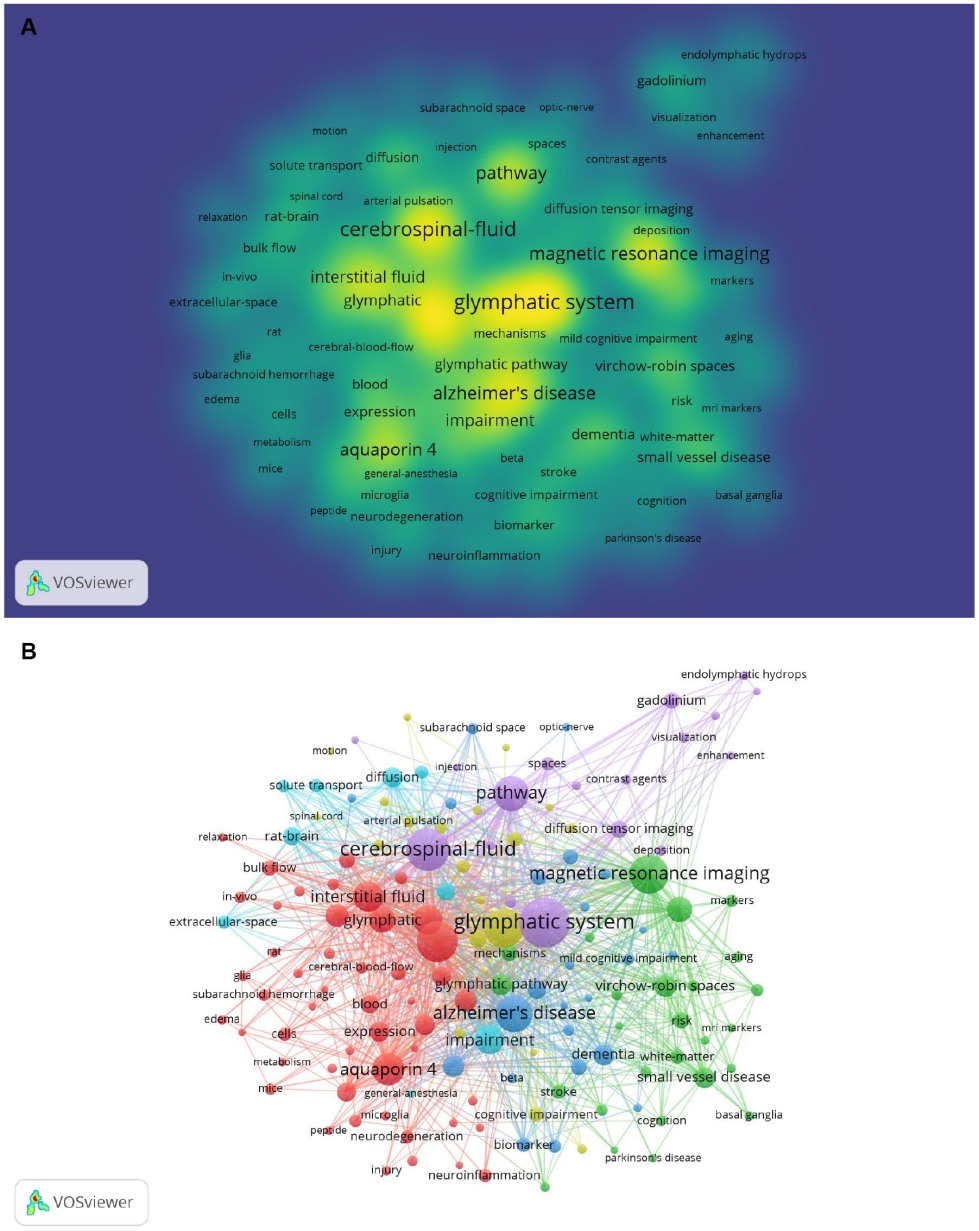
**(A)** The co-occurrence density map of keywords related to GS research. Minimum number of occurrences of keywords ≥5: **(B)** The co-occurrence network and clusters of keywords related to GS research. Minimum number of occurrences of keywords ≥5.

VOSviewer was used to perform network clustering analysis on keywords (Figure 9). By defining a number of keyword occurrences greater than or equal to 5 as the extraction threshold and combining keywords with the same meaning a total of 146 keywords were extracted. The number of links was 3,609 and the total link strength was 9,775. A total of 6 clusters with different colors were obtained representing 6 different research directions and ranges (Supplementary Table S3). The largest cluster was Cluster 1 (red) followed by Cluster 2 (green) Cluster 3 (blue) Cluster 4 (yellow) Cluster 5 (purple) and Cluster 6 (light blue). Cluster 1 contained 49 keywords in total for example “clearance” “system” “aquaporin 4” and “interstitial fluid.” There were 26 keywords in Cluster 2 such as “magnetic resonance imaging” “virchow-robin spaces” “perivascular spaces” and “small vessel disease.” Cluster 3 consisted of 24 keywords in all including “Alzheimers-disease” “dementia” “amyloid beta” and “disease.” Cluster 4 consisting of 20 keywords in total including “brain” “sleep” “flow” and “cognitive impairment.” Cluster 5 contained 18 keywords including “glymphatic system” “cerebrospinal-fluid” “pathway” and “gadolinium.” There were 9 entries in Cluster 6 such as “diffusion” “transport” “extracellular-space” and “impairment”

**Figure 10:**
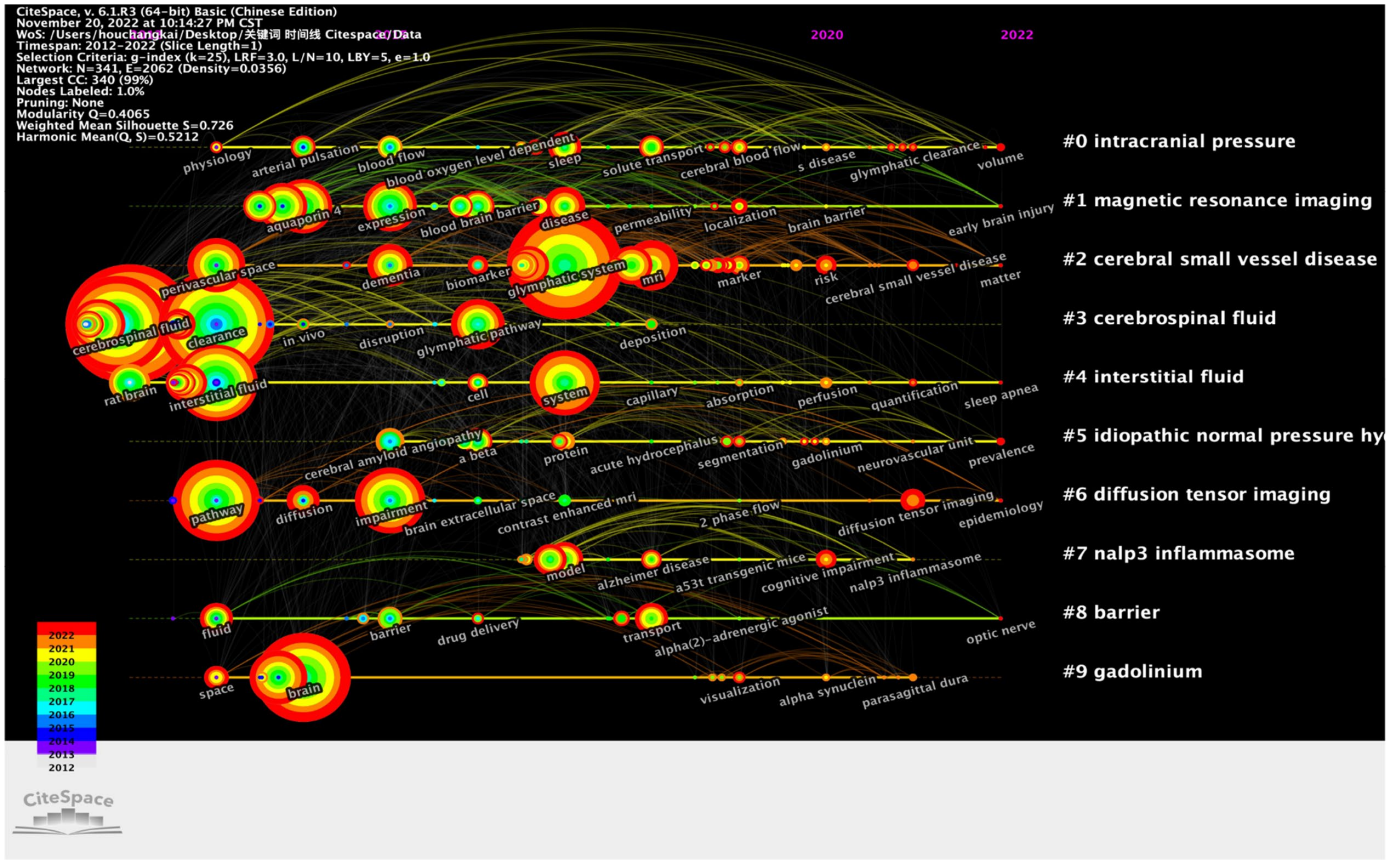
CiteSpace visualization map of timeline viewer related to the GS.

The timeline was helpful to explore the evolutionary track and stage characteristics of the research field. The timeline chart can cluster keywords and take time into account so that we can easily view the different periods of a specific topic in a certain research field. Additionally, it helped us explore the evolution track of the field. The research focus and evolutionary track of the GS research field at each stage are displayed in Figure 10.

## Discussion

### General information

This study aims to explore the research trends and hotspots in the GS field from 2012 to 2022 through visualization software. An increasing trend of GS research officially started in 2012. At that time, Iliff et al. first proposed the concept of the GS (Iliff et al., 2012). In the first 4 years, the number of studies on GS grew slowly, but during this period, the exploration of the basic structure and function of GS, as well as the construction of concepts, were extremely important. A series of original studies published by scholars led by Iliff, Nedergaard, Benveniste, and others during this period have continued to this day. It is still the paper that should be read and studied first in the GS field. In 2015, Professor Nedergaard M published a comprehensive review of the “The Glymphatic System: A Beginner’s Guide” on *Neurochemical Research* (Jessen et al., 2015), providing a detailed introduction to GS, which was very helpful for beginners and caused a great response. Subsequently, the phenomenon of increased publication volume became more apparent. The reason for this is that after a period of research, GS has gradually gained recognition from scholars. The important role GS plays in the physiological and pathological aspects of the nervous system has shown scholars its future development prospects; On the other hand, the progress of GS research methods and the expansion of research fields have greatly promoted the development of this process. According to the current trend, publication output will continue to grow positively. This suggests that the GS will continue to receive attention in the field of neuroscience in the future.

The publication of papers not only showed a dynamic trend over time but also a very characteristic distribution of countries and institutions. Most of the top 10 published articles were from developed countries. With the substantial investment in scientific research in recent years, China has obtained considerable returns. As the number of papers published by China in this field increased sharply in 2019, China surpassed Japan and became the country with most number of papers published in this field second only to the United States. The United States demonstrated its leading position in terms of the output of publications and international cooperation, far ahead of the rest in the GS field. Among the top 10 institutions, Univ Rochester from the United States was in an absolute leading position in terms of the number of documents and centrality, which indicated that the United States was the most influential country in this field, and their relevant research was far ahead of that of other countries. Denmark, Japan and Norway all had leading universities in the top 10. It is worth noting that Zhejiang University, Nanjing Medical University and Sun Yat Sen University from China were among the top 10 research institutions, which indicates that the GS has attracted extensive attention in China and the GS research field has made certain achievements. In the future, more researchers from China will be engaged in the GS field. And China can be considered for international cooperation in the next few years.

The top 10 journals listed in Table 2 were considered to be the core journals of GS publications. Most of these journals were located in the Q1 JCR region, which indicated that GS original research received great attention in the global scientific field. In the future, more studies on the GS will be published in these journals. Moreover, all the top 10 journals with the most citations were located in the Q1 JCR region, including Science, Science Translational Medicine and other high-level journals, which not only indicated people’s recognition of the research on the GS but also implied the status of these articles in the GS field. Simultaneously, these results also indicated that many high-quality and high-impact journals were very interested in GS-related research. It should be noted that Journal of Cerebral Blood Flow and Metabolism, Journal of Neuroscience and Neuroimage ranked among the top 10 in terms of the number of published articles and citations. This suggests their great influence in this field. In general, our research results will help researchers quickly and accurately find appropriate journals to obtain the latest progress or publish articles in the GS field. Combined with the summary of the GS field in Figure 2 and the double map superposition of the public cited journals in Figure 4, it was not difficult to see that the system was currently being transferred to clinical translation and the updating of imaging examination means.

Professor Nedergaard M was the most productive author. Professor Iliff JJ was co-cited the most among all co-cited authors, and had original research in various periods. They can be regarded as ideal role models.

Detecting the top keyword co-occurrence in the specific field could benefit the research guidelines and future directions. Our results showed that “glymphatic system,” “cerebrospinal fluid,” “clearance,” “brain” and “magnetic resonance imaging” were the most frequently used keywords, representing the main direction of original research in this field. They also echoed the current main GS research fields. After the network clustering analysis of the keywords, a total of 6 clusters were obtained. They were “glymphatic system,” “Alzheimers-disease,” “impairment,” “clearance,” “brain” and “magnetic resonance imaging “, representing the key directions of the 6 clusters. From the perspective of time classification, there were certain changes in keywords over time. Gradually, the main research interests moved toward the direction of imaging examination of the GS, which highlighted scholars’ exploration of noninvasive examination and the clinical translation of the GS.

### Knowledge base

Co-citation analysis is a research method used to measure the degree of correlation between papers. A knowledge base is a collection of commonly cited references. In this study, a total of 11 papers related to the GS field were included, which were co-cited most frequently as follows:

Martin Kaag Rasmussen et al. published “The glymphatic pathway in neurological disorders” in *Lancet Neurology* in 2018, which was the most cited paper (88 citations) (Rasmussen et al., 2018). This paper reviewed the structure and function of the GS under physiological conditions and imaging diagnostic methods and focused on the expression and regulation of the GS under different pathological conditions. The second most co-cited paper “Glymphatic MRI in idiopathic normal pressure hydrocephalus,” was published in *Brain* by Geir Ringstad et al. in 2017 (Ringstad et al., 2017). In this study, the authors evaluated the function of the GS in patients with idiopathic normobaric hydrocephalus and patients in a healthy control group after intrathecal injection of contrast agent. The results suggested that the function of the GS in patients with hydrocephalus was weakened. Moreover, the article provided a reference for MRI to be used in the measurement of human GS function in clinical diseases. The third most co-cited paper, “Flow of cerebrospinal fluid is driven by arterial pulsations and is reduced in hypertension,” was published in *Nature Communications* by Humberto Mestre and others in 2018 (Mestre et al., 2018b). In this study, the author used particle tracking to quantify the cerebrospinal fluid flow rate in the PVS of living mice. They found that the cerebrospinal fluid flow was mainly driven by the cardiac cycle. An increase in blood pressure will reduce the net flow in the PVS. Perfusion fixation will change the normal flow direction, resulting in a 10-fold reduction in PVS size. Benjamin T Kress et al. published the fourth most co-cited paper in *Annals of Neurology* in 2014 titled “Impairment of paravascular clearance pathways in the aging brain” (Kress et al., 2014). The aim of this study was to assess whether the efficiency of CSF-ISF exchange and interstitial solute clearance in the aging brain was impaired. The study found that an increase in age was related to a significant decrease in exchange efficiency between the subarachnoid cerebrospinal fluid and the brain parenchyma. In addition, the brain parenchyma of young mice and aged mice was injected with amyloid-β protein, and the amyloid-β clearance rate of the aged mice was 40% less than that of the young mice. It was suggested that impairment of the clearing function of the GS may lead to a decline in cognitive ability in elderly individuals, which may be a new target for the treatment of neurodegenerative diseases. Humberto Mestre et al. published the fifth most co-cited paper “Aquaporin-4-dependent glymphatic solute transport in the rodent brain “in *Elife* in 2018 (Mestre et al., 2018a). This meta-analysis explored the effect of Aqp4 knockout (KO) on the tracer transport of cerebrospinal fluid and interstitial fluid (ISF). Through retrospective analysis of previous studies, it was found that the CSF inflow of wild-type mice was higher than that of four different Aqp4 KO strains of mice and a strain of mice lacking perivascular Aqp4 (Snta1 KO). It was concluded that the tracer transport of KO mice and rats was significantly reduced. The sixth most co-cited paper was published in *NEUROCHEMICAL RESEARCH* by Nadia Aalling Jessen et al. in 2015 (Jessen et al., 2015). This review summarizes GS basic structural elements, organization, regulation, and functions. It also discussed the interactions between glymphatic functions and various diseases. Antoine Louveau et al. published the seventh most cocited paper in *Nature* in 2015 (Louveau et al., 2015). This article found that there were functional lymphatic vessels in the dural sinus, which can carry the fluid and immune cells in the cerebrospinal fluid and connect with the deep cervical lymph nodes. This changed the traditional concept of the central nervous system lacking a classic lymphatic drainage system and provided new clues for the etiology of neuroinflammation and neurodegenerative diseases related to immune system dysfunction. The eighth most co-cited paper was published by Benjamin A Plog et al. in the *Annual Review of pathology* in 2018 (Plog and Nedergaard, 2018). This review summarizes the role of the glymphatic pathway in the physiology of the central nervous system, the known factors regulatin glymphatic flow, and the pathological process related to the occurrence and development of diseases due to the disruption of glymphatic CSF-ISF exchange. Simultaneously, important directions in future research were also discussed. Geir Ringstad et al. published the ninth most co-cited paper in *JCI Insight* in 2018 (Ringstad et al., 2018). This study demonstrated that substances administered intrathecally can enter all human brain subregions. It was found that the elimination of trace substances was delayed in the dementia cohort. These observations translation the previous findings in animal studies into clinical studies and led to new prospects for intrathecal treatment, extravascular contrast-enhanced MRI and the assessment of brain clearance function. Toshiaki Taoka et al. published the tenth most co-cited paper in the *Japanese Journal of Radiology* in 2017 (Taoka et al., 2017). This study used a diffusion-based technique called diffusion tensor imaging analysis along the perivascular space (DTI-ALPS) to evaluate the activity of the human GS in AD patients. The results showed that the low diffusivity in DTI-APLS seemed to reflect the GS damage. It was found that this method can be used to evaluate GS activity. The 11th most cocited paper, “Impairment of glymphatic pathway function promotes tau pathology after traumatic brain injury,” was published in the *Journal of Neuroscience* by Iliff JJ et al. in 2014 (Iliff et al., 2014). The results suggested that the chronic impairment of glymphatic pathway function after brain trauma may be the key factor that leads to the brain being vulnerable to tau aggregation and neurodegeneration.

The top 10 co-cited reference with the highest average number of citations per year and the top 10 most frequently co-cited reference are highly overlapping. The 3 different documents are, respectively, the “Glymphatic failure as a final common pathway to dementia” published by Nedergaard M et al. in *Science* in 2020 (Nedergaard and Goldman, 2020), “Increased glymphatic influx is correlated with high EEG delta power and low heart rate in mice under anesthesia” published by Hablitz LM et al. in *Science Advances* in 2019 (Hablitz et al., 2019), and “Perivascular spaces in the brain: anatomy, physiology and pathology” published by Wardlaw JM et al. in *Nature Reviews. Neurology* in 2020 (Wardlaw et al., 2020). These three articles proposed the relationship between GS and EEG through original research, and analyzed the anatomy, physiological and pathological structure of GS as well as its potential relationship with neurodegenerative diseases through retrospective research.

In general, the 11 most frequently co-cited papers and the top 10 papers with an average number of citations per year were mostly from Q1 journals, with high recognition in GS field. These included not only high-quality reviews in the GS field but also relevant original research. The focus of these studies covered the discovery of the general regularity of the GS, the translation from animal experiments to clinical studies, and imaging research in this field.

### The analysis of hotspots and emerging topics

Frequently cited references refer to the sudden increase in the number of references in a certain period, which can help us find new topics and hotspots that attract much attention in a certain field. This study included the 47 most cited articles. The paper with the highest citation burst (intensity: 25.45) was “A paravascular pathway facilitates CSF flow through the brain parenchyma and the clearance of interstitial solutes, including amyloid β” published by Iliff JJ et al. in *Science Translational Medicine* in 2012 (Iliff et al., 2012). This study proposed that interstitial metabolic wastes can be transferred via paravascular pathways. AQP4 at astrocyte feet plays a crucial role. It was the document that established the concept of the GS and thus opened up this important research direction in the field of neuroscience. There were 7 papers with more than 10 citation bursts (Iliff et al., 2012, 2013a,b, 2014; Xie et al., 2013; Kress et al., 2014; Jessen et al., 2015), including 6 papers published by Professor Nedergaard M as corresponding author and 4 papers published by Professor Iliff JJ as the first author, which showed that Professor Nedergaard M and Professor Iliff JJ made outstanding contributions to this field. It should be noted that Thomas Misje Mathiisen published the paper entitled “The perivascular astroglial sheath provides a complete covering of the brain microvessels: an electron microscopic 3D reconstruction” in *Glia*. in 2010 (Mathiisen et al., 2010). This was 2 years earlier than the concept of the GS was proposed. This article has been cited many times, which indicates that the concept of the GS was not established overnight but developed slowly on the basis of previous theories. Meanwhile, 6 articles were still in a state of citation outbreak. Most of these articles were new works or reviews in the GS field, including the imaging evaluation of the GS and the role of the GS in neuroimmunology, cerebrovascular disease, and cognitive disorders (Brown et al., 2018; Louveau et al., 2018; Taoka and Naganawa, 2018; Ma et al., 2019; Mortensen et al., 2019; Zou et al., 2019).

According to the above analysis, the following important information can be obtained: a: The relevant high-quality and high-impact research of the GS mainly focuses on the regulation and functional changes of the physiological and pathological state and the imaging evaluation; b: Researchers tried to explain the occurrence and development of Alzheimer’s disease, cerebrovascular disease, brain injury and other related diseases with the relevant knowledge of GS, and tried to intervene; c: The GS still needs great progress, and the main researchers are concentrated in European and American countries; d: From the cluster analysis of key words (Figure 9), we can see that the lymphoid system has a high research enthusiasm in Alzheimer’s disease, and GS may be a potential target for improving Alzheimer’s disease; e: Meanwhile，according to the research, we predict that neuroimmunity will be associated with GS in the future, and may cause a small research upsurge.

On the basis of this study, our viewpoint is that more theoretical research is needed in the following directions: a: AQP4 is extremely important for GS, but the degree to which the polarity and expression level of AQP4 affect GS function is still unclear. The mechanism of AQP4 polarity changes needs further research, and the effective regulatory mechanisms for AQP4 are not yet abundant; b: The role of GS in the occurrence and development of neuroimmunity, especially the role of perivascular spaces in the transport of immune cells and immune substances; c: Is the GS function of different brain regions specific; d: How to safely translate GS research into clinical practice? At the same time, we propose a conjecture: whether it is possible to evaluate the whole brain GS function through the development of more effective and safe experimental and imaging technologies, in order to explore the potential predictive and therapeutic effects of GS function in proteinopathies, and promote the transformation of GS research from fundamental research to clinical work.

## Conclusion

In conclusion, since the concept of the GS was put forward in 2012, it has been rapidly verified and widely recognized in the past 10 years. Our results suggest that future research on the GS may focus on imaging evaluation, the role of the GS in different disease subtypes, and its role in neuroimmunity, which would help to provide a reference for future research directions.

## Data availability statement

The original contributions presented in the study are included in the article/Supplementary material, further inquiries can be directed to the corresponding authors.

## Author contributions

CH: conception and design, data analysis and interpretation, and manuscript writing. BW and XF: collection and assembly of data, data analysis, and interpretation. QL, HZ, WW, and JL: collection of data and data analysis. WR, XY, PW, and GZ: conception and design, administrative support, manuscript writing, and final approval of the manuscript. All the authors have read and approved the final content of this manuscript.

## Conflict of interest

The authors declare that the research was conducted in the absence of any commercial or financial relationships that could be construed as a potential conflict of interest.

## Publisher’s note

All claims expressed in this article are solely those of the authors and do not necessarily represent those of their affiliated organizations, or those of the publisher, the editors and the reviewers. Any product that may be evaluated in this article, or claim that may be made by its manufacturer, is not guaranteed or endorsed by the publisher.

## Footnotes

^1^http://bibliometric.com/
