# Supplementary material for: A bibliometric and knowledge-map analysis of the glymphatic system from 2012 to 2022
Source: Front Mol Neurosci. 2023 Aug 28;16:1148179. doi: 10.3389/fnmol.2023.1148179 (PMC10493282; doi:10.3389/fnmol.2023.1148179)
Supplement: Supplementary file 1 [file Table_1.docx]

| Rank | Centrality | Country/Region | Count | Rank | Centrality | Institution | Count |
| --- | --- | --- | --- | --- | --- | --- | --- |
| 1 | 0.44 | USA | 187 | 1 | 0.30 | Univ Rochester（USA） | 44 |
| 2 | 0.27 | ENGLAND | 25 | 2 | 0.18 | Massachusetts Gen Hosp（USA） | 8 |
| 3 | 0.23 | GERMANY | 16 | 3 | 0.14 | Keio Univ（JAPAN） | 3 |
| 4 | 0.21 | PEOPLES R CHINA. | 123 | 4 | 0.11 | Yale Sch Med（USA） | 11 |
| 5 | 0.21 | SCOTLAND | 9 | 5 | 0.11 | Sun Yat Sen Univ（CHINA） | 12 |
| 6 | 0.13 | SWEDEN | 20 | 6 | 0.10 | Nagoya Univ（JAPAN） | 22 |
| 7 | 0.09 | FINLAND | 10 | 7 | 0.09 | Univ Oslo（NORWAY） | 22 |
| 8 | 0.07 | DENMARK | 29 | 8 | 0.07 | Univ Edinburgh  (UK) | 5 |
| 9 | 0.06 | CANADA | 13 | 9 | 0.07 | Univ Copenhagen（DENMARK） | 26 |
| 10 | 0.04 | NETHELANDS | 8 | 10 | 0.07 | Nanjing Med Univ（CHINA） | 12 |

Supplementary Table1:Top 10 countries/institutions in terms of centrality.
