# Supplementary material for: A bibliometric and knowledge-map analysis of the glymphatic system from 2012 to 2022
Source: Front Mol Neurosci. 2023 Aug 28;16:1148179. doi: 10.3389/fnmol.2023.1148179 (PMC10493282; doi:10.3389/fnmol.2023.1148179)
Supplement: Supplementary file 2 [file Table_2.docx]

| **Rank** | **Year** | **Author** | **Title** | **Journal** | **Citation/years** | **Centrality** |
| --- | --- | --- | --- | --- | --- | --- |
| 1 | 2018 | Martin Kaag Rasmussen | The glymphatic pathway in neurological disorders | Lancet Neurology | 17.6 | 0.01 |
| 2 | 2018 | Humberto Mestre | Flow of cerebrospinal fluid is driven by arterial pulsations and is reduced in hypertension | Nature communication | 13.6 | 0.02 |
| 3 | 2018 | Humberto Mestre | Aquaporin-4-dependent glymphatic solute transport in the rodent brain | Elife | 13.4 | 0.03 |
| 4 | 2017 | Geir Ringstad | Glymphatic MRI in idiopathic normal pressure hydrocephalus | Brain | 12.2 | 0.11 |
| 5 | 2018 | Benjamin A Plog | The Glymphatic System in Central Nervous System Health and Disease: Past, Present, and Future | Annual Review Of Pathology | 11.4 | 0.02 |
| 6 | 2020 | Nedergaard M | Glymphatic failure as a final common pathway to dementia | Science | 11.3 | 0.02 |
| 7 | 2018 | Geir Ringstad | Brain-wide glymphatic enhancement and clearance in humans assessed with MRI | JCI Insight | 11.2 | 0.02 |
| 8 | 2019 | Hablitz LM | Increased glymphatic influx is correlated with high EEG delta power and low heart rate in mice under anesthesia | Science advances | 10 | 0.20 |
| 9 | 2020 | Wardlaw JM | Perivascular spaces in the brain: anatomy, physiology and pathology | Nature Reviews. Neurology | 9.3 | 0.01 |
| 10 | 2017 | Toshiaki Taoka | Evaluation of glymphatic system activity with the diffusion MR technique: diffusion tensor image analysis along the perivascular space (DTI-ALPS) in Alzheimer's disease cases | Japanese Journal of Radiology | 9 | 0.05 |

Supplementary Table2:According to the number of times the literature is cited each year, the top 10 co-cited literature related to the field of Glyphatic System.
