# Supplementary material for: A bibliometric and knowledge-map analysis of the glymphatic system from 2012 to 2022
Source: Front Mol Neurosci. 2023 Aug 28;16:1148179. doi: 10.3389/fnmol.2023.1148179 (PMC10493282; doi:10.3389/fnmol.2023.1148179)
Supplement: Supplementary file 3 [file Table_3.docx]

| **Cluster** | **Keywords** |
| --- | --- |
| 1（49） | activation、amyloid-beta peptide、aquaporin 4、astrocytes、barrier、blood、blood-brain-barrier、brain interstitial fluid、bulk flow、cells、central-nervous-system、cerebral-blood-flow、choroid-plexus、clearance、deep cervical lymph、deletion、disruption、drainage、edema、expression、glia、glymphatic、hippocampus、inflammation、injury、interstitial fluid、in-vivo、ischemia、lymphatic、lymphatics、metabolism、mice、microglia、model、mouse model、neurodegeneration、neuroinflammation、peptide、perfusion、permeability、protein、rat、rats、relaxation、subarachnoid hemorrhage、system、t-cells、traumatic brain injury、volume |
| 2（26） | age、aging、basal ganglia、blood-pressure、cerebral small vessel disease、cognition、cognitive function、dysfunction、enlarged perivascular spaces、glymphatic pathway、ischemic-stroke、magnetic resonance imaging、markers、mechanisms、memory、mri markers、parkinson's disease、perivascular spaces、risk、segmentation、small vessel disease、stroke、vascular dementia、virchow-robin spaces、white-matter、white-matter hyperintensities |
| 3（24） | alzheimer's disease、amyloid、amyloid beta、association、basement-membranes、beta、biomarker、cerebral amyloid angiopathy、dementia、disease、drug delivery、fluid、mild cognitive impairment、normal-pressure hydrocephalus、optic-nerve、pathology、perivascular drainage、pet、pressure、prevalence、risk-factors、stiffness、subarachnoid space、tau |
| 4（20） | alpha-synuclein、arterial pulsation、blood-flow、brain、cerebrospinal fluid (csf)、cerebrospinal-fluid flow、cognitive impairment、dynamics、flow、fluctuations、glymphatic clearance、head-injury、hydrocephalus、intracranial-pressure、motion、physiology、quantification、s disease、sleep、spinal cord |
| 5（18） | cerebrospinal-fluid、contrast agents、deposition、diffusion tensor imaging、endolymphatic hydrops、enhancement、gadolinium、glymphatic function、glymphatic system、idiopathic normal pressure hydrocephalus、images、injection、localization、menieres-disease、pathway、spaces、visualization、water |
| 6（9） | anesthesia、contrast-enhanced mri、diffusion、extracellular-space、general-anesthesia、impairment、rat-brain、solute transport、transport |

Supplementary Table3:A total of 146 keywords were classified by 6 clusters.
